# Adaptation and feasibility assessment of a school-based Suicide Prevention Intervention for Adolescents (SPREAD Study) in Nigeria: protocol for a cluster randomised controlled feasibility trial

**DOI:** 10.1136/bmjopen-2026-124182

**Published:** 2026-10-01

**Authors:** Abiodun O Adewuya,, Olushola Olibamoyo,, Olabisi E Oladipo, Olayinka Atilola, Adesegun Fatusi, Ife O Abodunrin, Kemi Akeju

**Affiliations:** 1 Department of Behavioural Medicine, Lagos State University College of Medicine, Ikeja, Nigeria; 2 Department of Social Welfare Services, Lagos State University Teaching Hospital, Lagos, Lagos, Nigeria; 3 Centre for Mental Health Research and Initiative, Lagos, Nigeria; 4 Insititute of Public Health, College of Health Sciences, Obafemi Awolowo University, Ife, Osun, Nigeria; 5 Department of Educational Foundations and Counselling Psychology, Lagos State University, Ojo, Nigeria; 6 Department of Economics, Ekiti State University, Ado Ekiti, Nigeria

**Keywords:** Adolescents, Africa South of the Sahara, Suicide & self-harm, Psychosocial Intervention, Schools

## Abstract

**Introduction:**

Adolescent suicide constitutes a critical public health challenge in low-income and middle-income countries (LMICs), where structural deficits in mental health infrastructure compound an already substantial burden. In Nigeria, where suicidal ideation among secondary school students has been documented at a lifetime prevalence of 22.9%, curative clinic-based responses are neither scalable nor sustainable. School-based universal interventions offer a pragmatic upstream alternative. The Youth Aware of Mental Health (YAM) programme has demonstrated robust efficacy in European contexts, reducing incident suicide attempts by approximately 50%; however, its transferability to the West African educational and cultural environment remains untested. This protocol describes a study designed to culturally adapt YAM for Nigerian secondary schools and assess the feasibility of conducting a definitive cluster randomised controlled trial in Lagos State.

**Methods and analysis:**

This study employs a hybrid type 1 effectiveness–implementation design, structured as a two-arm parallel-group cluster randomised feasibility trial across 10 public secondary schools in Lagos State. Approximately 600 adolescents aged 13–17 years will be recruited. Prior to the trial, YAM will be systematically adapted using the Method for Programme Adaptation through Community Engagement framework, producing the YAM-Nigeria curriculum. Schools will be randomised to receive either the culturally adapted YAM-Nigeria programme (five sessions of 45–60 min each delivered by trained external facilitators) or enhanced usual practice (standard health education curriculum supplemented with suicide awareness materials). Primary feasibility outcomes include school recruitment rates (target ≥60%), student consent rates (target ≥70%), retention at the 3-month follow-up (target ≥75%), implementation fidelity (target ≥80% adherence) and acceptability (target ≥70% student satisfaction). Secondary exploratory outcomes include suicidal ideation, depression, mental health literacy, stigma and help-seeking behaviour, measured to estimate the SD and intraclass correlation coefficient required to power a future definitive trial. Progression to a definitive phase III trial will be governed by pre-specified traffic light criteria.

**Ethics and dissemination:**

Ethical approval has been granted by the Lagos State University Teaching Hospital Health Research Ethics Committee (Ref: LREC/06/10/2656) and the Liverpool School of Tropical Medicine Research Ethics Committee (Ref: RGETEM045). A two-stage consent process requiring written parental consent and adolescent assent will be implemented. Findings will be disseminated through open-access peer-reviewed publications, policy briefs for the Lagos State Ministries of Health and Education and plain-language summaries for participating communities.

**Trial registration number:**

ISRCTN registry, ISRCTN69846459. Registered on 24 November 2025.

**STRENGTHS AND LIMITATIONS OF THIS STUDY**

- The cluster randomised feasibility design reflects the school-level mode of intervention delivery and pre-specifies progression criteria across six implementation domains (recruitment, retention, fidelity, dose, acceptability and safety) providing an explicit, evidence-based decision framework for advancement to a definitive trial.
- Systematic cultural adaptation of the Youth Aware of Mental Health programme using the Method for Programme Adaptation through Community Engagement framework, guided by a multidisciplinary steering committee including adolescents and educators, is completed prior to trial commencement, reducing the risk of type III error.
- The hybrid type 1 effectiveness–implementation design enables simultaneous assessment of implementation feasibility and preliminary clinical parameter estimation within a single study, maximising the utility of the research investment.
- The sample of 600 students across 10 schools is calculated to estimate key feasibility parameters with adequate precision and generate the intraclass correlation coefficient required for powering a definitive trial but is not powered to detect statistically significant differences in rare clinical outcomes such as suicide attempts.
- Restriction to public co-educational secondary schools in urban Lagos limits transferability of findings to rural settings, Northern Nigeria and the private school sector, where sociodemographic and infrastructural contexts differ considerably.

## Introduction

### Background and rationale

### Global and regional epidemiology of adolescent suicide

 Suicide mortality among young people constitutes a public health crisis of considerable magnitude, currently ranking as the second leading cause of death among adolescents aged 10–19 years globally.^1 2^ While often framed within the discourse of high-income nations, the burden of this ‘silent epidemic’ falls disproportionately on low-income and middle-income countries (LMICs), where approximately 90% of the world’s adolescents reside and where >75% of global suicides occur.^3 4^ The developmental trajectory from suicidal ideation to attempts frequently crystallises during adolescence and young adulthood,^5^ a critical window wherein underlying psychological vulnerabilities intersect with emerging social pressures, academic stressors and identity formation.

In sub-Saharan Africa, this crisis is compounded by systemic scarcity of mental health infrastructure and a paucity of reliable surveillance data.^6^ Nigeria, as the continent’s most populous nation with a youth demographic exceeding 40 million, exemplifies these challenges. Recent epidemiological data from Lagos State document a lifetime prevalence of suicidal ideation at 22.9% and suicide attempts at 8.3% among secondary school students^7^: figures that align with or exceed rates observed in many high-income settings. These statistics likely underestimate the true burden, obscured by the profound stigma associated with mental illness and legal statutes that continue to criminalise attempted suicide.^8^ The interplay of these factors creates a climate of silence, rendering traditional help-seeking pathways inaccessible or fraught with legal and reputational risk.

#### Treatment gap and imperative for upstream prevention

The capacity of the Nigerian healthcare system to respond to this burden is severely constrained. With <300 psychiatrists serving a population >200 million, the ratio of mental health professionals to citizens is critically insufficient,^9^ and specialised child and adolescent mental health services are virtually non-existent outside a handful of tertiary centres in major urban hubs.^10^ This structural deficit produces a vast treatment gap in which the overwhelming majority of adolescents experiencing psychological distress receive no professional care.^11^

Given these resource constraints, reliance on curative, clinic-based models is neither scalable nor sustainable. The public health imperative has consequently shifted toward upstream prevention—universal interventions designed to bolster protective factors and socio-emotional resilience across entire populations.^12 13^ Schools have emerged as the optimal ecological niche for such strategies, offering unparalleled access to the adolescent demographic within a naturalistic setting that avoids the stigmatisation often associated with psychiatric facilities.^14 15^ Curriculum-based programmes that enhance mental health literacy, promote adaptive coping strategies and destigmatise help-seeking are increasingly viewed as the preferred approach in LMICs, circumventing the resource intensity and potential intrusiveness of individual screening protocols.^16–18^ In a systematic review, Doty *et al*^17^ identified youth aware of mental health (YAM) as one of only a small number of school-based suicide prevention programmes with evidence from LMICs, underscoring both the relevance of the current study and the size of the evidence gap it aims to address.

### Intervention: youth aware of mental health

Among existing school-based interventions, YAM stands as a leading evidence-based candidate.^17 19^ Developed in Sweden, YAM is a manualised, universal intervention that integrates cognitive behavioural therapy principles with experiential learning.^20^ Unlike didactic approaches, it relies heavily on interactive role play and open dialogue, empowering adolescents as active participants in their mental health literacy.^21 22^ Clinical efficacy was established in the Saving and Empowering Young Lives in Europe study (a multicentre cluster randomised controlled trial (cRCT) involving over 11 000 adolescents across 10 countries) where YAM significantly reduced incident suicide attempts by approximately 50% and severe suicidal ideation by 50% at the 12-month follow-up.^20^ Subsequent implementations in the USA and Australia have reinforced these findings.^23–25^, ^26^

#### Implementation gap: risks of uncritical transfer

While the empirical support for YAM is robust in Western contexts, its applicability in the Nigerian educational and cultural landscape remains unverified.^17 27^ The global mental health field is well acquainted with the risks of ‘lift and shift’ implementation, where psychosocial interventions are transplanted from high-income settings to LMICs without sufficient scrutiny of their cultural congruence.^27 28^ Cultural narratives surrounding mental health in Nigeria are frequently intertwined with spiritual causality and communal reputation, differing substantially from the biomedical and individualistic models that underpin most Western interventions.^4 6^ The educational environment in Lagos public schools (characterised by large class sizes, rigid didactic traditions and variable infrastructure) differs markedly from the European settings where YAM was originally conceived.^29 30^ Furthermore, the direct engagement with topics of suicide and depression required by YAM may encounter resistance from parents or religious stakeholders.^6 31^

Implementing YAM without rigorous adaptation and feasibility testing risks type III error: evaluating an intervention that was never delivered as intended or one that failed to engage its target population due to cultural mismatch.^27 28^ Adherence to the UK Medical Research Council (MRC) framework for developing and evaluating complex interventions^32^ is therefore essential, given that this framework mandates a phased, iterative trajectory that prioritises context over rigid standardisation.

### Theoretical framework and study design

To address these complexities, the Suicide PREvention for School ADolescents (SPREAD study) employs a hybrid type 1 effectiveness–implementation design.^33^ This methodological architecture allows simultaneous evaluation of implementation feasibility, the primary focus, alongside preliminary clinical signals. The study is anchored in the Method for Programme Adaptation through Community Engagement (M-PACE),^34^ which guides the pre-trial adaptation phase, and the Reach, Effectiveness, Adoption, Implementation, Maintenance (RE-AIM framework),^35^ which structures the feasibility assessment. By systematically addressing the know-do gap, SPREAD aims to generate a scalable, culturally grounded suicide prevention toolkit suitable for integration into the Nigerian school curriculum.

### Study aim and objectives

The primary aim is to culturally adapt YAM and assess the feasibility, acceptability and implementation parameters of conducting a definitive cRCT in Lagos, Nigeria.

#### Primary feasibility objectives

- **Recruitment capability:** to estimate school recruitment rates and student consent rates against pre-defined progression criteria (target: ≥60% for schools; ≥70% for students).
- **Retention viability:** to determine attrition at 3 months post-intervention within the often-transient inner-city school population of Lagos (target: ≥75%).
- **Implementation fidelity:** to evaluate the extent to which YAM-Nigeria can be delivered as intended by trained non-school facilitators within public secondary schools (target: ≥80% adherence to core components).
- **Acceptability:** to explore how the intervention interacts with local norms regarding mental health and pedagogy, assessed through stakeholder interviews and quantitative measures (target: ≥70% student satisfaction).
- **Barriers and facilitators:** to identify contextual determinants of implementation success or failure using the Consolidated Framework for Implementation Research.^36^

#### Secondary exploratory objectives

- **Parameter estimation:** to estimate the SD and intraclass correlation coefficient (ICC) for primary clinical outcome measures (suicidal ideation and depression scores), critical for powering a future definitive trial.
- **Preliminary effectiveness:** to observe trends in suicidal ideation, depression and stigma at 3 months post-intervention as proof of concept.
- **Health economics:** to establish preliminary cost-of-delivery data per student and resource use, informing future budget impact analyses.

## Methods and analysis

### Study design

The SPREAD study employs a hybrid type 1 effectiveness–implementation design,^33^ structured as a two-arm, parallel-group, cluster randomised feasibility trial. The unit of randomisation is the school (cluster) rather than the individual student, necessitated by the classroom-based, collective learning mechanism of YAM.^20^ Individual randomisation within the same classroom would introduce substantial contamination risk, as students inevitably share experiences and materials between sessions. Cluster randomisation also mirrors the real-world mechanism of educational policy implementation, where curriculum decisions are made at the institutional level. The flow of participants through the trial is illustrated in figure 1.

**Figure 1:**
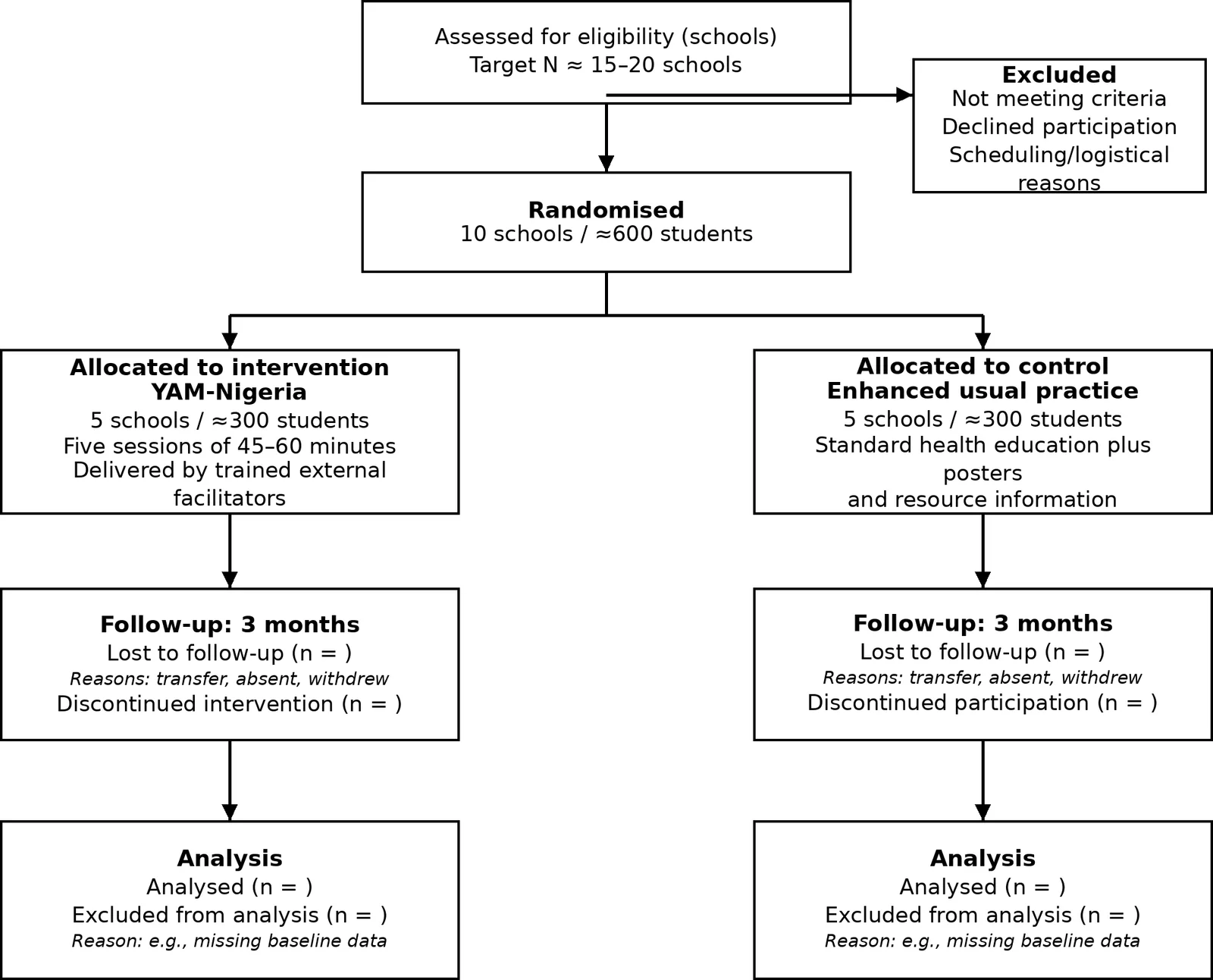
CONSORT flow diagram for the SPREAD feasibility study. The diagram illustrates participant flow from school identification through to the 3-month follow-up assessment, including enrolment, allocation, intervention delivery and analysis. CONSORT, Consolidated Standards of Reporting Trials; SPREAD, Suicide PREvention for School ADolescents.

### Study setting

The study is situated within the public secondary school system of Lagos State, Nigeria. Lagos, a hyper-urbanised megalopolis with a population exceeding 20 million, represents a critical testing ground for adolescent health interventions owing to its high population density and marked socio-economic stratification. The state maintains an extensive network of over 300 government-owned secondary schools. Participating schools will be purposively sampled from urban and peri-urban districts to ensure a representative cross-section of the educational landscape. These institutions are typically characterised by large class sizes (frequently exceeding 40 students), limited mental health infrastructure and a student body drawn predominantly from lower-income and middle-income families. The language of instruction is English, although Yoruba and other indigenous languages predominate in domestic settings.

### Eligibility criteria

#### School-level eligibility

A comprehensive summary is provided in table 1. Schools must be public, co-educational secondary schools recognised by the Lagos State Ministry of Education; have a minimum enrolment of 600 students; possess at least six distinct classes for students aged 13–17 years (each containing a minimum of 20 students); provide documented willingness of the principal to participate via a Memorandum of Understanding (MoU); and have a private classroom space suitable for confidential role-play sessions. Schools currently implementing other structured suicide prevention programmes, undergoing scheduled major infrastructure works or leadership transitions, or concurrently participating in other research projects will be excluded.

**Table 1:** Eligibility criteria for schools (clusters) and students

Level	Inclusion criteria	Exclusion criteria
School level	Governance: public, co-education al secondary schools in Lagos State. Size: minimum enrolment of 600 students. Demographics: at least six distinct classes for ages 13–17 years (minimum of 20 students per class). Consent: documented agreement from principal via MoU. Infrastructure: availability of private space for group activities and role plays.	Competing interventions: current implementation of other suicide prevention programmes. Instability: scheduled major transitions (relocations, leadership changes and construction) during the study period. Research fatigue: concurrent participation in other research projects.
Student level	Enrolment: currently enrolled in selected classes. Age: aged 13–17 years at baseline. Language: sufficient English proficiency to engage with intervention materials. Consent: written parental consent and adolescent assent.	Cognitive capacity: severe impairment preventing group participation. Safety risk: active psychotic symptoms requiring immediate clinical evacuation. *Note: suicidal ideation is NOT an exclusion criterion; these students are managed via the Safety Protocol*. *3. Discipline****:*** current suspension or expulsion precluding attendance.

MOM, Memorandum of Understanding.

#### Student-level eligibility

Inclusion requires current enrolment in selected classes, age 13–17 years at baseline, sufficient English proficiency to engage with intervention materials and written parental consent alongside written adolescent assent. Students with severe cognitive impairment precluding group participation, those presenting with active psychotic symptoms requiring immediate clinical management or those under current suspension or expulsion are excluded. Notably, students expressing suicidal ideation are not excluded but are managed via the study’s safety escalation protocol: excluding them would introduce a systematic bias against the very population the intervention aims to support.

### Interventions

#### Cultural adaptation phase (Method for Programme Adaptation through Community Engagement framework)

Prior to the feasibility trial, the original YAM curriculum will undergo systematic adaptation using the M-PACE framework.^34^ This process distinguishes between the intervention’s deep structure (core mechanisms such as role play, peer-to-peer learning and non-judgmental stance), which must be preserved, and its surface structure (language, names, scenarios and visual materials), which must be contextualised for the Nigerian setting.^29^ An Adaptation Steering Committee comprising researchers, YAM developers, adolescents and educators will guide iterative cycles of theatre testing and stakeholder feedback to produce the YAM-Nigeria toolkit (figure 2).

**Figure 2:**
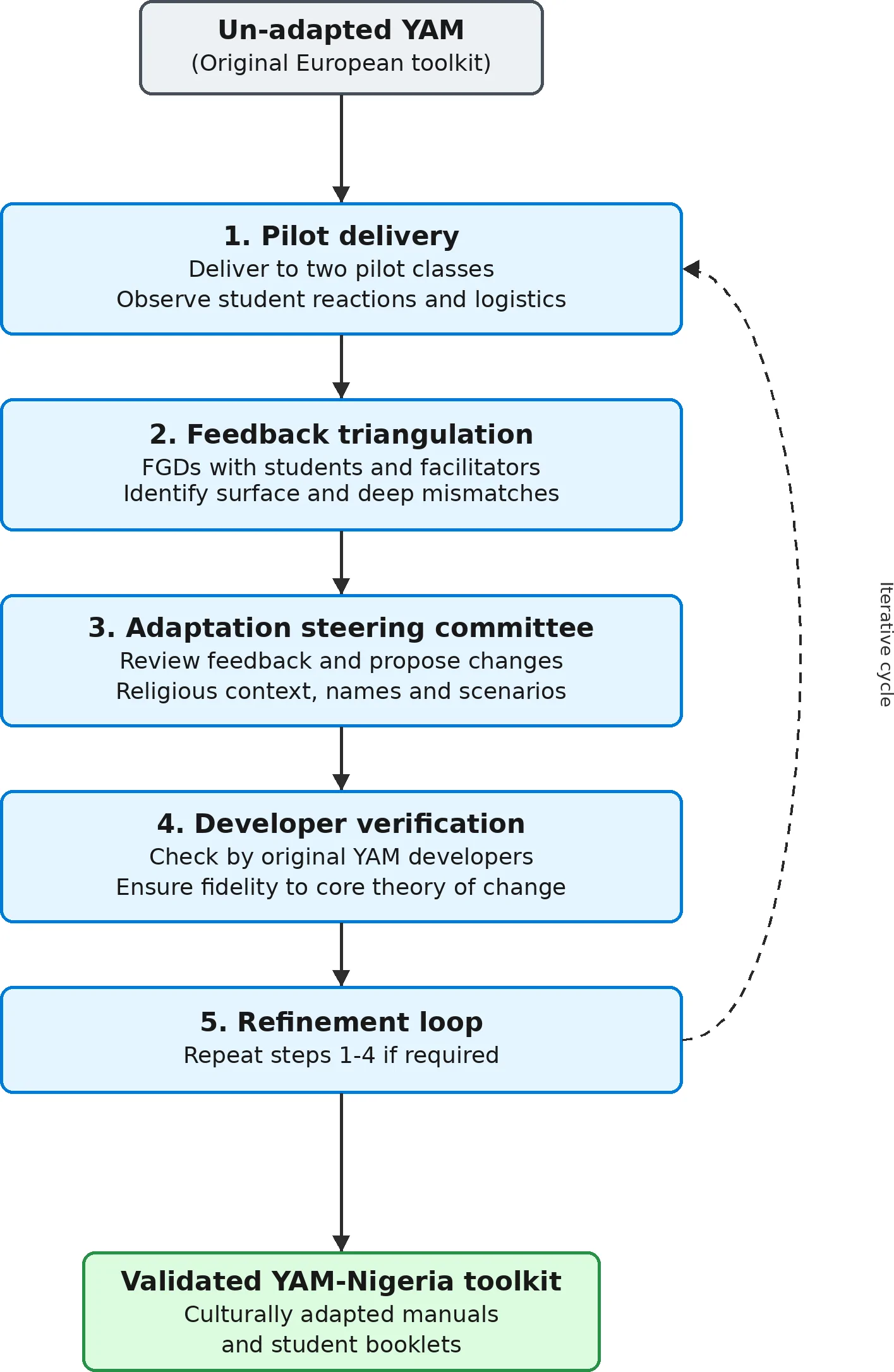
M-PACE adaptation framework for YAM-Nigeria. The figure illustrates the iterative stages of the Method for Programme Adaptation through Community Engagement, from community needs assessment through systematic adaptation to the production of the culturally contextualised YAM-Nigeria toolkit. FGDs, focus group discussions; YAM, Youth Aware of Mental Health.

#### Intervention arm: YAM-Nigeria

Schools allocated to the intervention arm will receive the culturally adapted YAM programme: a universal, manualised intervention merging cognitive behavioural therapy principles with experiential learning. The curriculum consists of five sessions, each lasting 45–60 min, delivered over 3–5 weeks by trained external facilitators (psychology graduates and social workers) rather than school staff. This arrangement mitigates the rigid teacher–student hierarchies common in Nigerian classrooms, creating a psychological safety environment that encourages students to discuss sensitive topics without fear of academic or disciplinary repercussion.

- Session 1 introduces mental health as a spectrum using the health/unwell metaphor, with dilemma cards prompting critical thinking about everyday stressors.
- Sessions 2 and 3 involve progressive role play exploring crisis scenarios, depression recognition and peer support: all scenarios adapted to reflect Nigerian realities such as academic pressure and religious expectations.
- Session 4 consolidates learning through repeated role play or thematic discussions.
- Session 5 focuses on resource mapping, enabling students to identify trusted adults and local services and develop personal help-seeking action plans

Students receive an adapted YAM booklet, and classrooms are furnished with posters reinforcing the validity of help-seeking.

#### Control arm: enhanced usual practice

Control schools continue with the standard Lagos State health education curriculum, which addresses general hygiene and disease prevention but lacks mental health-specific pedagogy. An attention-effect control enhancement comprises high-quality suicide awareness posters and resource cards listing local support services, ensuring all participants have access to crisis information while preserving the ability to isolate the specific impact of the YAM pedagogical mechanism. Consistent with the principles of distributive justice, control schools will be offered the full YAM-Nigeria intervention after trial completion.

### Outcomes

#### Primary feasibility outcomes

The trial’s success will be judged against progression criteria derived from the RE-AIM framework.^35^

- Recruitment (reach) will be assessed as the proportion of eligible schools agreeing to participate (target≥60%) and the proportion of eligible students providing consent and assent (target≥70%).
- Retention (adoption) will be measured as the proportion of enrolled participants completing the 3-month follow-up assessment (target≥75%).
- Implementation fidelity will be assessed via facilitator checklists and audio recordings, measuring both adherence (proportion of core YAM components delivered as intended, target≥80%) and dose (student attendance across five sessions, target≥75% attending≥4 sessions).
- Acceptability will be assessed using the Acceptability of Intervention Measure (AIM) and post-trial qualitative interviews (target≥70% rating the intervention as acceptable).

#### Secondary exploratory outcomes

Clinical and process outcomes will be measured at baseline (T0) and 3 months post-intervention (T1). These are not powered for significance testing but are collected to estimate parameters for future power calculations. A full breakdown of outcome measures and assessment timepoints is provided in table 2.

**Table 2:** Summary of outcome measures and assessment timepoints

Outcome domain	Instrument/metric	Description/target	T0	T1
Primary feasibility				
Recruitment (reach)	Enrolment logs	School rate, ≥60%; student rate, ≥70%	✓	
Retention	Follow-up logs	Proportion completing the 3-month assessment (≥75%)		✓
Fidelity	Observation checklist	Proportion of core YAM content delivered as intended (≥80%)	During intervention	
Adherence (dose)	Attendance register	Student attendance rate across five sessions (≥75%)	During intervention	
Acceptability	Satisfaction survey/AIM	Student satisfaction scores (≥70% rating ‘satisfied’)		✓
Data feasibility	Data quality logs	Completeness of outcome measures (≥85% item completion)	✓	✓
Implementation	Qualitative interviews	Barriers and facilitators via CFIR/RE-AIM constructs		✓
Secondary clinical				
Suicidal behaviour	Paykel Suicide Scale	Frequency of suicidal ideation, plans, and attempts (past 3 months)	✓	✓
Depression and anxiety	RCADS-25	Symptoms of depression and anxiety (Revised Child Anxiety and Depression Scale, 25-item)	✓	✓
Mental health literacy	MHLS	General knowledge about mental health, treatment options, and help-seeking (Mental Health Literacy Scale)	✓	✓
Suicide literacy	LOSS (short form)	Knowledge about suicide causes, warning signs, and prevention (Literacy of Suicide Scale)	✓	✓
Stigma of suicide	SOSS	Stigmatising attitudes towards suicide (Stigma of Suicide Scale)	✓	✓
Attitudes toward suicide	ATS	General attitudes toward suicide (Attitudes Toward Suicide Scale)	✓	✓
Self-esteem	RSES	Global self-worth (Rosenberg Self-Esteem Scale)	✓	✓
Help-seeking	GHSQ/AHSQ	Intentions to seek help vs actual help-seeking behaviour	✓	✓
Health economics	CSRI (adapted)	Health service use and delivery costs	✓	✓

AHSQ, Actual Help-Seeking Questionnaire; AIM, Acceptability of Intervention Measure; ATS, Attitudes Toward Suicide Scale; CFIR, Consolidated Framework for Implementation Research; CSRI, Client Service Receipt Inventory; GHSQ, General Help-Seeking Questionnaire; LOSS, Literacy of Suicide Scale; MHLS, Mental Health Literacy Scale; RCADS-25, Revised Child Anxiety and Depression Scale; RE-AIM, Reach, Effectiveness, Adoption, Implementation, Maintenance; RSES, Rosenberg Self-Esteem Scale; SOSS, Stigma of Suicide Scale; T0, baseline (pre-randomisation); T1, follow up (3 months post-intervention); YAM, Youth Aware of Mental Health.

Suicidal behaviour will be assessed using the Paykel Suicide Scale,^37^ depression and anxiety using the Revised Child Anxiety and Depression Scale,^38^ general mental health literacy using the Mental Health Literacy Scale,^39^ suicide-specific literacy using the Literacy of Suicide Scale,^40^ stigmatising attitudes toward suicide using the Stigma of Suicide Scale,^41^ help-seeking intentions and behaviour using the General Help-Seeking Questionnaire^42^ and Actual Help-Seeking Questionnaire^43^ and health service use using an adapted Client Service Receipt Inventory.^44^

### Participant timeline

The study timeline follows the Standard Protocol Items: Recommendations for Interventional Trials (SPIRIT) schedule.^45^ During months 1–3 (enrolment), schools are recruited, site visits conducted and consent materials distributed. Baseline assessment (T0) precedes randomisation, after which YAM-Nigeria is delivered during months 3–5 (intervention arm) alongside poster installation in control schools. Three-month follow-up assessment (T1) is administered at month 8. Process evaluation via qualitative interviews and fidelity monitoring occurs throughout the intervention phase. The complete schedule of enrolment, interventions and assessments is presented in table 3.

**Table 3:** Schedule of enrolment, interventions and assessments (SPIRIT schedule)

	Enrolment (months 1–3)	Allocation (month 3)	Intervention (months 3–5)	Follow-up (month 8/T1)
Enrolment				
Eligibility screen (schools)	X			
Informed consent (parents)	X			
Assent (students)	X			
Cluster randomisation		X		
Interventions				
YAM-Nigeria Programme (5 sessions)			X	
Enhanced usual practice (control)			X	
Assessments				
Socio-demographics	X			
Recruitment and reach rates	X			
Retention (attrition rates)				X
Fidelity (checklists and observation)			X	
Acceptability (AIM Scale)				X
Suicidality (Paykel Suicide Scale)	X			X
Depression/anxiety (RCADS-25)	X			X
Mental health literacy (MHLS)	X			X
Suicide literacy (LOSS)	X			X
Stigma (SOSS) and attitudes (ATS)	X			X
Self-esteem (RSES)	X			X
Help-seeking (GHSQ/AHSQ)	X			X
Resource use and costs (CSRI)	X			X
Qualitative interviews				X
Safety monitoring (adverse events)			X	X

AHSQ, Actual Help-Seeking Questionnaire; AIM, Acceptability of Intervention Measure; ATS, Attitudes Toward Suicide Scale; CSRI, Client Service Receipt Inventory; GHSQ, General Health-Seeking Questionnaire; LOSS, Literacy of Suicide Scale; MHLS, Mental Health Literacy Scale; RCADS-25, Revised Child Anxiety and Depression Scale ; SOSS, Stigma of Suicide Scale; SPIRIT, Standard Protocol Items: Recommendations for Interventional Trials.

### Sample size

Formal power calculations to detect a minimum clinically important difference are not appropriate for this feasibility protocol; the sample size is instead predicted on the precision of estimation for key feasibility parameters. We aim to recruit 10 schools (clusters) with approximately 60 students per school, yielding a total of approximately 600 participants. With this sample, we can estimate a recruitment rate of 60% with a 95% CI width of approximately ±4%. The sample is also sufficient to generate a reliable ICC estimate for suicidal ideation (a parameter currently absent from the West African literature^7 17^), which is essential for calculating the cluster-adjusted sample size of a future definitive trial, since underestimating the design effect is a recognised cause of underpowered cRCTs.^46^ The target of 10 clusters is consistent with published guidance on minimum cluster numbers for feasibility trials of this design.^47^

### Recruitment

A multi-stage purposive sampling strategy will be employed in collaboration with the Lagos State Ministry of Education, selecting schools to represent diverse socio-economic catchments. Initial engagement will involve high-level stakeholder meetings followed by site visits to secure the principal’s commitment via an MoU. Student recruitment will use information sheets and consent forms distributed to all eligible students in selected classes; town hall-style meetings will be held for parents to explain the study verbally, addressing literacy barriers. A two-stage consent process will require written informed consent from a parent or guardian, followed by written assent from the adolescent. Trained research team members will confirm consent and assent before baseline assessment. Example parent/guardian informed consent and adolescent assent forms are provided in online supplemental file 4.

### Allocation and blinding

Randomisation will occur at the school level to prevent contamination. An independent statistician will generate the allocation sequence using a computer-generated random number list, stratified by school size (above or below median enrolment) to ensure balance. Allocation will be concealed using sequentially numbered, opaque, sealed envelopes, opened only after baseline data collection is complete and the school’s participation is irrevocably confirmed. Owing to the nature of the behavioural intervention, it is not possible to blind students or school staff to their allocation. Research assistants collecting follow-up data will be blinded to school allocation where feasible, and analysts will work with coded datasets.

### Data collection methods

Quantitative data will be collected via Research Electronic Data Capture (REDCap)^48^ hosted on a secure, firewalled server at LASUCOM. To mitigate internet connectivity issues common in Lagos schools, the REDCap mobile app will be installed on project-dedicated tablets, enabling offline data entry with encrypted synchronisation. Paper questionnaires will serve as contingency backup, digitised within 48 hours via double-data entry. Qualitative data from interviews and focus group discussions (FGDs), conducted using stakeholder-specific topic guides (online supplemental file 2), will be recorded using encrypted digital voice recorders; audio files will be transferred to the secure server immediately on return to the research office and deleted from recording devices after confirmed transfer. Transcription will be performed by the research team, removing direct identifiers during the process.

### Data management

All participants will be assigned a unique alphanumeric study ID on enrolment. The link log connecting study IDs to personal names and contact details will be stored separately from clinical data on a password-protected, encrypted server accessible only to the principal investigator and study coordinator. Access to the REDCap database will be governed by least-privilege principles: data entry staff will have add/edit access only, with no export rights; statisticians will access de-identified datasets only. Automated logic within REDCap will enforce range checks and mandatory fields for critical safety questions.

### Statistical methods

The analytical strategy reflects the feasibility status of this trial: the primary focus is precision of estimation rather than hypothesis testing, and the primary outputs of interest are the ICC and SD of clinical outcome measures rather than p values. All quantitative analyses will follow the intention-to-treat principle.

Feasibility outcomes will be reported using descriptive statistics with 95% CIs, assessed against pre-defined progression criteria. A 95% CI that excludes the target threshold will trigger a review of implementation strategies. Exploratory clinical outcomes will be analysed using generalised linear mixed models to account for the hierarchical structure of the data (students nested within classes and nested within schools), with random intercepts for schools and fixed effects for study arm, baseline scores and relevant covariates. A detailed analysis of missingness patterns will precede any imputation strategy; where data are deemed missing at random, multiple imputation will be employed.

Qualitative data from key informant interviews and FGDs with students, teachers and parents will be analysed using the framework method,^49^ a matrix-based approach suited to applied policy research. Analysis will proceed through five stages: familiarisation, thematic framework development, indexing, charting, and mapping and interpretation. A joint display technique will integrate quantitative and qualitative findings. Progression to a definitive phase III trial will be governed by the pre-specified traffic light criteria presented in table 4.

**Table 4:** Pre-specified progression criteria for advancement to definitive trial

Domain	Feasibility metric	Green (go)	Amber (amend)	Red (stop)
Recruitment (reach)	School adoption: proportion of eligible schools agreeing to participate	≥60%	40%–59%	<40%
	Student consent: proportion of eligible students providing consent/assent	≥70%	50%–69%	<50%
Retention (adoption)	Follow-up rate: proportion of enrolled participants completing the 3-month follow-up	≥75%	60%–74%	<60%
Implementation	Fidelity: proportion of core YAM content delivered as intended (via checklist)	≥80%	60%–79%	<60%
	Adherence (dose): proportion attending ≥4 of 5 sessions	≥75%	50%–74%	<50%
Acceptability	Satisfaction: proportion rating intervention as ‘satisfactory’ or higher	≥70%	50%–69%	<50%
Safety	Adverse events: incidence of SAEs directly attributable to intervention participation	None	Minor/managed (protocol revision required)	Pattern of harm (termination)

SAE, serious adverse event; YAM, Youth Aware of Mental Health.

### Implementation fidelity assessment

Implementation fidelity will be assessed via the Facilitator Fidelity and Competency Checklist (online supplemental file 1) completed by independent observers during random session audits. Observers will rate both adherence to core components (target≥80%) and competence of delivery. The distribution of dose received (number of sessions attended per student) will be examined to identify patterns of engagement.

### Safety monitoring

Since the study involves universal screening, some students with previously unidentified active suicidality will be identified. A risk-stratified safety escalation protocol (online supplemental file 3) will be active throughout. REDCap will run a custom daily script during data collection to flag any record indicating active suicidal ideation on Paykel items 4 or 5, triggering an automated alert to the study clinical officer.

The triage system operates on three levels. At low risk (ideation expressed but no plan or active intent), a trained research assistant debriefs the student privately and provides signposting to the school counsellor. At moderate risk (ideation with a plan but no immediate intent), the study clinical officer conducts a same-day private assessment, a safety plan is co-created with the student and parents are notified unless doing so poses a clear risk of harm to the child. At high risk (active intent, recent attempt or access to lethal means), the student is never left alone; the principal investigator and on-call psychiatrist are contacted immediately; and if parents are unavailable or uncooperative, the student is escorted by two study staff to the emergency psychiatric unit at LASUTH or the Federal Neuropsychiatric Hospital, Yaba.

An independent Data and Safety Monitoring Committee (DSMC) comprising a senior psychiatrist, a biostatistician and an ethicist will meet at months 1, 6 and 10 to review aggregate safety data. The DSMC holds authority to recommend trial termination if evidence emerges that the intervention is causing harm.

### Study organisation and governance

The Trial Steering Committee, chaired by an independent expert, provides overall supervision on behalf of the sponsor (LASUCOM) and funder (UKRI), approving the protocol, monitoring progress against milestones and reviewing final deliverables. A Project Advisory Board (PAB) comprising representatives from the Lagos State Ministry of Education, Parents–Teachers Association and youth representatives meets quarterly to advise on recruitment strategies and the cultural appropriateness of dissemination materials.

### Patient and public involvement

Patient and public involvement was embedded from the outset of SPREAD. Adolescents with lived experience of school-related distress participated in preliminary workshops that informed the decision to adapt YAM rather than a screening-based programme and that shaped the emphasis on help-seeking, stigma and culturally recognisable stressors as secondary outcomes. Youth and parent representatives sit on the PAB and contribute to the design of participant information sheets, assent materials and the cultural framing of recruitment (resilience and life skills rather than suicide alone). They will advise on recruitment strategies and the acceptability of session scheduling and content during the trial. The burden of the five-session classroom intervention was reviewed with adolescent and educator advisers during the M-PACE adaptation phase (theatre testing and stakeholder feedback) and will be reassessed through post-session acceptability measures and qualitative interviews. Study findings will be shared with participating schools, parents and students through plain-language summaries framed around emotional well-being and resilience; youth representatives on the PAB will co-interpret qualitative findings and help shape community-facing outputs.

### Ethics and dissemination

#### Ethical considerations

Ethical approval has been secured from the Lagos State University Teaching Hospital Health Research Ethics Committee (Ref: LREC/06/10/2656) and the Liverpool School of Tropical Medicine Research Ethics Committee (Ref: RGETEM045). A two-stage consent process is mandatory: written informed consent from parents or guardians, followed by written assent from the adolescent. Example parent/guardian informed consent and adolescent assent forms are provided in online supplemental file 4. Students are explicitly informed that they may withdraw from the research components while continuing to participate in the educational sessions, preventing educational exclusion. To encourage honest reporting, all survey data are de-identified using unique study IDs; however, limits to confidentiality are explicitly stated in consent forms, specifying that disclosure of imminent harm to self or others triggers mandatory reporting.

### Dissemination plan

The dissemination plan targets three distinct audiences. For the scientific community, the full protocol and subsequent results will be published in open-access peer-reviewed journals, with findings presented at regional and international conferences. For policymakers, concise policy briefs will be produced for the Lagos State Ministries of Health and Education, highlighting cost implications and scalability factors. For community stakeholders, plain-language summaries will be distributed to participating schools, parents and community leaders and framed around resilience and emotional well-being rather than focusing solely on suicide prevention. After publication of the primary trial results, a suitably de-identified participant-level dataset, data dictionary and statistical analysis code will be archived in a recognised research data repository that provides persistent identifiers and controlled-access functionality. Given the sensitivity of data concerning minors and suicidality, participant-level data will not be openly downloadable; access will require review of a data request, execution of a Data Use Agreement and any required ethical approvals, in accordance with participant consent, applicable data-protection requirements and sponsor and funder policies. The repository identifier and access conditions will be reported in the primary results publication.

## Discussion

### Bridging the know–do gap in global mental health

SPREAD addresses a critical juncture in the globalisation of evidence-based psychiatry. While the burden of adolescent suicide is disproportionately concentrated in LMICs, the vast majority of preventive interventions have been developed and validated in the resource-rich contexts of the Global North.^17 50^ This discrepancy produces a profound know–do gap: we know that school-based universal interventions such as YAM can reduce suicide attempts by up to 50% in European settings,^20^ yet we do not know whether comparable effects are achievable in the complex, resource-constrained and culturally distinct environment of Nigerian public education. This protocol moves decisively beyond simplistic lift-and-shift approaches by rigorously testing the conditions under which evidence-based prevention might succeed.^27 28^ The study challenges the assumption that Western pedagogical models privileging open dialogue and individual agency will automatically resonate in a setting where adult–child hierarchies are rigid and mental distress is frequently interpreted through spiritual or moral frameworks.^6 30^

### Methodological strengths and innovations

A primary strength of this protocol is its strict adherence to the MRC framework for developing and evaluating complex interventions,^32^ treating the implementation context as an active variable rather than background noise. The hybrid type 1 design^33^ is a strategic innovation that allows simultaneous interrogation of implementation barriers (such as school scheduling rigidities and parental consent refusal) while gathering preliminary clinical signals, thereby maximising research efficiency and accelerating the pathway to a definitive trial.

The integration of M-PACE^34^ ensures that YAM adaptation is systematic rather than ad hoc, rigorously distinguishing between the intervention’s deep structure (core mechanisms) and surface structure (cultural references, language and scenarios). Engagement of a PAB that includes ministry officials, parents and adolescents from the outset fosters local ownership that is essential for long-term sustainability. The cluster randomised design is methodologically vital, mitigating the substantial contamination risks inherent in school settings and ensuring the distinction between intervention and control conditions remains valid.

### Navigating contextual challenges

The operationalisation of this protocol directly addresses structural realities of the Nigerian educational system. Recognising that public school teachers face overwhelming workloads and large class sizes, the protocol mandates trained external facilitators rather than burdening existing staff.^51^ Beyond fidelity considerations, this decision creates a psychological safety environment that may be more conducive to sensitive disclosure outside the school’s authority hierarchy.^52^ The study also anticipates and mitigates the profound stigma surrounding suicide in Nigeria, where suicidal behaviour remains criminalised.^53^ The dual-consent mechanism respects family authority while preserving adolescent autonomy, and recruitment materials are carefully framed around resilience, stress management and life skills rather than focusing exclusively on suicide prevention.

### Limitations

Several limitations intrinsic to the feasibility scope of this study merit acknowledgement. The sample of 600 adolescents across 10 schools, while rigorously justified for estimating recruitment and retention parameters with precision, is not powered to detect statistically significant differences in rare events such as suicide attempts; any clinical signals in the secondary outcomes should therefore be interpreted as exploratory and hypothesis-generating rather than confirmatory. The 3-month follow-up window is sufficient for assessing immediate acceptability, safety and short-term retention but precludes evaluation of long-term behavioural maintenance or the sleeper effects sometimes observed in psychosocial interventions^20 24^; future definitive trials will require a 12-month follow-up period. The inability to blind students and facilitators to allocation introduces a potential performance bias, a common challenge in behavioural trials mitigated here through self-report outcome measures and blinding of outcome assessors where feasible. Finally, restriction to public secondary schools in Lagos State means that findings may not generalise fully to rural settings, Northern Nigeria or the private school sector, where resources, cultural norms and demographics differ substantially.

### Implications for policy and practice

If the progression criteria are met, the immediate output will be a validated, culturally adapted YAM-Nigeria toolkit ready for large-scale evaluation, providing the Lagos State Ministry of Basic and Secondary Education with the first empirical evidence base for integrating mental health promotion into the secondary school curriculum. The study will also generate the ICC for suicidal ideation in this population: a parameter that is currently non-existent for West African schools and is essential for calculating the sample size of a future definitive trial. Conversely, if the study identifies insurmountable implementation barriers, it will yield equally important evidence, preventing the wasteful scaling of an incompatible intervention and redirecting policy efforts toward alternative modalities.

## Trial status

The current protocol is Version 2.0, dated November 2025. Recruitment of schools is scheduled to commence in month 2 of the study timeline (February 2026), with student enrolment in month 3. Data collection is anticipated to be completed by Month 9.

## Supplementary material

10.1136/bmjopen-2026-124182 online supplemental file 1

10.1136/bmjopen-2026-124182 online supplemental file 2

10.1136/bmjopen-2026-124182 online supplemental file 3

10.1136/bmjopen-2026-124182 online supplemental file 4
