# Supplementary material for: Adaptation and feasibility assessment of a school-based Suicide Prevention Intervention for Adolescents (SPREAD Study) in Nigeria: protocol for a cluster randomised controlled feasibility trial
Source: BMJ Open. 2026 Oct 1;16(10):e124182. doi: 10.1136/bmjopen-2026-124182 (PMC13629916; doi:10.1136/bmjopen-2026-124182)
Supplement: Supplementary file 1 [file bmjopen-16-10-s001.pdf]

SUPPLEMENTARY FILE 1: FACILITATOR FIDELITY AND COMPETENCY CHECKLIST

SPREAD STUDY: YAM-NIGERIA FIDELITY CHECKLIST

Purpose: To be completed by independent observers during random session audits to ensure the intervention is delivered as intended (Implementation Fidelity).

Session Details

School ID	______________________________
Facilitator Name	______________________________
Class ID	______________________________
Observer Name	______________________________
Date	/__/______
Session Number	[ ] 1 [ ] 2 [ ] 3 [ ] 4 [ ] 5

Part A: Adherence (Content Delivery)

Did the facilitator cover the core components prescribed in the manual?

Core Component	Delivered?	Notes / Deviations
1. Opening: Clear introduction of session goals and safety rules (confidentiality, respect)	[ ] Yes [ ] No	
2. Concept Definition: Correctly defined key terms (e.g., Stress vs. Crisis, Depression) using adapted local examples	[ ] Yes [ ] No	
3. Materials: Dilemma Cards and booklets were distributed and used effectively	[ ] Yes [ ] No	
4. Interaction: Facilitated the main Role-Play activity for at least 20 minutes	[ ] Yes [ ] No	
5. Closing: Summarised key learning points and distributed resource/contact cards	[ ] Yes [ ] No	
6. Safety Check: Visually scanned room for distress and checked in with quiet students	[ ] Yes [ ] No	

Adherence Score: _______ / 6 core components delivered **= _______ %**

Part B: Competence (Quality of Delivery)

Rate the facilitator's skill on a scale of 1 (Poor) to 5 (Excellent).

Domain	Rating (1–5)	Observer Comments
**Psychological Safety:** Maintained a non-judgmental atmosphere; managed mockery or bullying immediately	1 2 3 4 5	
**Fidelity to Method:** Avoided lecturing or giving personal advice; encouraged student-led solutions	1 2 3 4 5	
**Engagement:** Encouraged participation from diverse students (not only the most vocal)	1 2 3 4 5	
**Cultural Resonance:** Used language and examples that resonated with the students (e.g., local context)	1 2 3 4 5	
**Crisis Management:** Handled sensitive disclosures (if any) calmly and according to protocol	1 2 3 4 5	

Competence Average: _______ / 5

Overall Assessment

[ ] **PASS** — All 6 Core Components delivered AND Competence average ≥ 3

[ ] **FAIL** — Retraining Required

If FAIL, specify reason: ____
