# Supplementary material for: Adaptation and feasibility assessment of a school-based Suicide Prevention Intervention for Adolescents (SPREAD Study) in Nigeria: protocol for a cluster randomised controlled feasibility trial
Source: BMJ Open. 2026 Oct 1;16(10):e124182. doi: 10.1136/bmjopen-2026-124182 (PMC13629916; doi:10.1136/bmjopen-2026-124182)
Supplement: Supplementary file 2 [file bmjopen-16-10-s002.pdf]

SUPPLEMENTARY FILE 2: QUALITATIVE TOPIC GUIDES

General Instructions for Interviewers

- **Safety First:** Before starting, remind the participant of the safety protocol. If a participant becomes distressed or discloses active suicidal intent, stop the recording immediately and activate the Safety Escalation Protocol (Supplementary File 3).
- **Consent:** Confirm permission to audio record before proceeding.
- **Tone:** Maintain a non-judgmental, curious stance throughout. There are no 'wrong' answers.

Topic Guide A: Student Focus Group Discussion (FGD)

Target Group: Students in the Intervention Arm (YAM) | **Duration:** 45–60 minutes

1. Introduction and Warm-up *(5 minutes)*

Goal: Build rapport and establish psychological safety.

- "Let's start by talking about school life in general. What are the biggest stress points for students in this school right now?"

2. Experience of the YAM Sessions *(Acceptability)*

- "You recently attended the YAM programme. What was your honest first impression of it?"
- "Which session do you remember most? Why?" *Probe: Was it the role-plays, the dilemma cards, or the group discussions?*
- "Did you feel comfortable acting out the stories? Why or why not?"
- "What did you think about the facilitators — the people who led the sessions? Did they feel different from your regular teachers?"

3. Cultural Relevance *(Appropriateness)*

- "The stories in the Dilemma Cards — did they feel like 'real life' for a student in Lagos, or did they feel foreign to you?"
- "Was there anything in the booklet or posters that felt strange or didn't make sense in our culture?"
- "How do you think your parents would feel if they read the student booklet?"

4. Impact and Help-Seeking *(Effectiveness / Reach)*

- "Since the programme finished, have you used any of the skills you learned? Can you give an example?"
- "If a friend told you they were feeling 'fed up with life,' would you know what to do now? How is that different from before the programme?"
- "Did anyone try to use the helpline numbers on the resource card? Was it easy to get through?"

5. Recommendations *(Maintenance)*

- "If we run this programme again next year, what is the **one thing** you would change?"

Topic Guide B: Teacher and School Administrator Interview (KII)

Target Group: Principals, Vice-Principals, School Counsellors | **Duration:** 30–45 minutes

1. Feasibility and Logistics

- "How difficult was it to fit the sessions into the school timetable? Were there major disruptions to normal teaching?"
- "Did the programme clash with examinations or other mandatory school activities?"
- "How was the communication with the research team? Did you feel adequately informed about what was happening at each stage?"

2. Perception of Impact *(Effectiveness)*

- "Did you notice any changes in the students' behaviour or interactions during or after the programme?" *Probe: Changes in discipline, openness to staff, or peer support behaviours.*
- "Have there been more students coming to report mental health concerns to staff since the programme? How have you handled those disclosures?"

3. Sustainability *(Adoption / Maintenance)*

- "Do you think this programme is something the school could continue to offer in the future without the research team?"
- "What would the school need to run this permanently — for example, funding, staff training, or a Ministry of Education policy directive?"
- "How did teachers feel about having external facilitators coming into their classrooms?"

Topic Guide C: YAM Facilitator Debrief (KII)
Target Group: Trained external facilitators delivering the YAM-Nigeria intervention | **Duration:** 45 minutes

1. Fidelity and Delivery

- "Which parts of the manual were easiest to deliver? Which were the hardest, and why?"
- "Did you ever feel you had to depart from the script to make students understand the material? Can you give a specific example?" *Probe for distinction between 'protocol drift' and legitimate 'cultural adaptation.'*

2. Classroom Dynamics

- "How did the students generally respond to the role-plays — were they shy, enthusiastic, or at times disruptive?"
- "Did you encounter any high-risk disclosures or situations during sessions? How did the safety protocol work in practice?"
- "Did the physical classroom environment (noise, heat, or space constraints) affect the quality of the sessions?"

3. Cultural Fit

- "Were there any concepts (for example, distinguishing depression from ordinary sadness) that required significant extra explanation?"
- "Did you feel the students trusted you as an 'outsider' to the school, or did your external status create barriers?"

Topic Guide D: Parent Interview (KII)
Target Group: Parents or guardians of participating students (purposive sample) | **Duration:** 30 minutes

1. Awareness and Consent

- "When you first received the letter describing a 'suicide prevention' study, what was your immediate reaction?" *Probe: Fear, relief, confusion, or religious concern?*
- "Was the information sheet clear? Did you feel you had sufficient time and information to make a decision?"

2. Observations

- "Did your child talk about the sessions at home? What did they say?"
- "Have you noticed any changes in the way your child handles stress or talks about their feelings since the programme?"

3. Acceptability

- "Do you think schools should be teaching children about mental health and suicide, or do you feel this is better addressed at home, at church, or at the mosque?"
