# Supplementary material for: Adaptation and feasibility assessment of a school-based Suicide Prevention Intervention for Adolescents (SPREAD Study) in Nigeria: protocol for a cluster randomised controlled feasibility trial
Source: BMJ Open. 2026 Oct 1;16(10):e124182. doi: 10.1136/bmjopen-2026-124182 (PMC13629916; doi:10.1136/bmjopen-2026-124182)
Supplement: Supplementary file 3 [file bmjopen-16-10-s003.pdf]

SUPPLEMENTARY FILE 3: SAFETY ESCALATION PROTOCOL (SUICIDALITY)

Purpose: A mandatory standard operating procedure (SOP) for all study staff governing the identification, triage, and management of students who express suicidal ideation or intent during the course of the study.

1. TRIGGER

This protocol is activated if a student discloses suicidal ideation or intent through any of the following routes:

- Responses to the Paykel Suicide Scale at baseline or follow-up assessment
- Verbal disclosure during a YAM session or group activity
- Report from a teacher, school counsellor, or peer

2. IMMEDIATE TRIAGE: The Traffic Light System

🟢 **GREEN — Low Risk** *Ideation expressed, but no specific plan and no active intent to act*

Step	Action
**Action**	Research Assistant (RA) debriefs the student privately after the session or assessment
**Resource**	Provide the student with a Help Card listing local helplines and crisis contacts; signpost to the school counsellor
**Follow-up**	RA checks in with the student at the next scheduled session or contact point
**Documentation**	Log incident in REDCap Safety Incident Register within 4 hours

🟡 **AMBER — Moderate Risk** *Ideation with a specific plan, but no immediate intent to act*

Step	Action
**Action**	RA alerts the Study Clinical Officer immediately; student is not left unsupervised
**Assessment**	Study Clinical Officer conducts a brief structured risk assessment in a private room
**Notification**	Parents or guardians are contacted by phone, unless doing so poses a clear risk of harm or abuse to the child
**Safety Plan**	A written safety plan is co-created with the student and retained on file
**Documentation**	Log incident in REDCap Safety Incident Register within 4 hours

🔴 **RED — High Risk** *Active intent to act, recent attempt, or confirmed access to lethal means*

Step	Action
**Immediate Action**	Student must **NEVER** be left alone from this point forward
**Escalation**	RA immediately contacts the Principal Investigator and On-Call Psychiatrist by phone
**Parental Contact**	Parents or guardians are summoned to the school immediately
**Referral**	If parents are unavailable or uncooperative, the student is escorted by **two** study staff members to the Emergency Psychiatric Unit at LASUTH or the Federal Neuropsychiatric Hospital, Yaba
**Reporting**	A Serious Adverse Event (SAE) form must be submitted to both ethics committees within **24 hours**
**Documentation**	Log incident in REDCap Safety Incident Register within 4 hours

3. DOCUMENTATION

All incidents, regardless of risk level, must be logged in the **Safety Incident Register** on REDCap within **4 hours** of the incident occurring. The log must include: date, school ID, student Study ID, risk classification, actions taken, persons notified, and outcome.

LASUTH = Lagos State University Teaching Hospital; RA = Research Assistant; SAE = Serious Adverse Event; SOP = Standard Operating Procedure.
