# Supplementary material for: Adaptation and feasibility assessment of a school-based Suicide Prevention Intervention for Adolescents (SPREAD Study) in Nigeria: protocol for a cluster randomised controlled feasibility trial
Source: BMJ Open. 2026 Oct 1;16(10):e124182. doi: 10.1136/bmjopen-2026-124182 (PMC13629916; doi:10.1136/bmjopen-2026-124182)
Supplement: Supplementary file 4 [file bmjopen-16-10-s004.pdf]

SUPPLEMENTARY FILE 4: EXAMPLE OF PARTICIPANT CONSENT AND ASSENT FORMS

Study: Adaptation and Feasibility Assessment of a School-Based Suicide Prevention Intervention for Adolescents in Nigeria (the SPREAD Study)

Trial registration: ISRCTN69846459 | Protocol version 2.0, November 2025

Note: These are example forms supplied for journal publication to illustrate the two-stage consent process described in the protocol. Operational study participation is governed by the ethics-approved consent and assent documents in use at study sites.

A. Example Parent/Guardian Informed Consent Form

Principal Investigator: Professor Abiodun O. Adewuya, Department of Behavioural Medicine, Lagos State University College of Medicine, Ikeja, Lagos, Nigeria

Email: abiodun.adewuya@lasucom.edu.ng

Ethics approvals: LASUTH HREC: LREC/06/10/2656; Liverpool School of Tropical Medicine REC: RGETEM045

Why is this study being done?
The SPREAD study is examining whether a culturally adapted school-based mental health and suicide-prevention programme can be delivered acceptably, safely, and feasibly in Lagos public secondary schools. The study is a feasibility trial designed to inform a later, larger trial.

What will my child be asked to do?
If you consent and your child assents, your child will complete a baseline research assessment before the school is randomly allocated to one of two study groups. Schools in the intervention group will receive the culturally adapted YAM-Nigeria programme, consisting of five 45–60 minute sessions delivered over approximately 3–5 weeks by trained external facilitators. Control schools will continue usual health education with enhanced suicide-awareness materials. A three-month follow-up research assessment will be completed. Some participants may later be invited to a separate qualitative interview or focus group; any additional consent or assent required for audio recording will be sought before that activity.

Are there risks or disadvantages?
Some questions and discussions concern depression, suicidal thoughts, stress, and help-seeking and may cause temporary discomfort or distress. Study staff are trained to respond to distress. If a student discloses imminent risk of harm to self or others, confidentiality cannot be maintained: the study safety procedure will be activated so that appropriate adults and clinical services can be involved to protect the student.

Are there benefits?
There may be no direct personal benefit. The study may improve understanding of how school-based mental health promotion and suicide-prevention programmes can be implemented safely and appropriately in Nigerian schools.

Is participation voluntary?
Yes. Participation in the research components is voluntary. You may decline permission or later withdraw your child from research assessments without penalty. A student who withdraws from the

research questionnaires may continue to participate in the educational sessions, as described in the study protocol. Information already collected up to the point of withdrawal may be retained for the integrity of the study unless removal is required by applicable ethics or data-protection rules.

How will information be protected?
Research data will be identified by a unique Study ID rather than by the student’s name. The link between names and Study IDs will be stored separately with restricted access. Reports and publications will not identify individual students. Any sharing or archiving of suitably de-identified research data will be governed by participant consent, ethics approval, data-protection requirements, and controlled-access procedures.

Consent statements

☐ I have read the participant information provided for this study, or it has been explained to me in a language I understand.

☐ I have had the opportunity to ask questions and have received satisfactory answers.

☐ I understand that participation in the research is voluntary and that I may withdraw my child from research participation without penalty.

☐ I understand that the school, rather than the individual student, will be randomly allocated after baseline assessment.

☐ I understand that the study includes questions and discussions about mental health and suicide that may be distressing.

☐ I understand the limits to confidentiality where there is an immediate risk of harm to my child or another person.

☐ I understand how my child’s research information will be kept confidential and reported without direct identifiers.

☐ I give permission for my child to participate in the SPREAD study research assessments and study activities described above.

Name of parent/guardian: ____

Name of student: ____

Signature/thumbprint of parent/guardian: __________________ Date: ____ / ____ / ______

Name/signature of person obtaining consent: ____________________________

Date: ____ / ____ / ______

B. Example Adolescent Assent Form (Ages 13–17 years)
This form is for students whose parent or guardian has given permission for them to be invited to the SPREAD study. You should only take part if you also want to.

What is the study about?
We are studying whether a school mental health programme can be used safely and acceptably in Lagos secondary schools. The programme teaches young people about mental health, coping with difficult situations, helping friends, and seeking support.

What will happen if I agree?
You will complete a questionnaire before your school is randomly placed in one of two groups. If your school receives YAM-Nigeria, there will be five sessions lasting about 45–60 minutes each over 3–5 weeks. If your school is in the comparison group, it will continue usual health education and receive enhanced suicide-awareness information. You will be asked to complete another research assessment about three months later.

Could anything make me uncomfortable?
Some questions or discussions are about stress, depression, suicidal thoughts, and getting help. You can tell a member of the research team if you feel upset. If you tell us that you are in immediate danger of harming yourself or someone else, we cannot keep that information secret. We will involve appropriate adults and health professionals to help keep you safe.

Do I have to take part?
No. It is your choice. You can say no even if your parent or guardian has given permission. You may also stop taking part in the research later. If you stop completing research questionnaires, you may still attend the educational sessions if you wish, as described in the protocol.

Will people know what I said?
Your research information will use a Study ID instead of your name. The research team will protect your personal information, and reports will not identify you. The main exception is if we believe you or someone else is in immediate danger, when we must act to help keep people safe.

Assent statements

- ☐ The study has been explained to me in words I understand.
- ☐ I have been able to ask questions.
- ☐ I understand that taking part in the research is my choice and I can stop later without punishment.
- ☐ I understand that my school will be randomly placed in one of two groups after the baseline assessment.
- ☐ I understand that some questions may be upsetting and that I can ask for help.
- ☐ I understand that information is private unless there is an immediate safety risk.
- ☐ I want to take part in the SPREAD study research.

Name of student: ____

Student signature: __________________________________ Date: ____ / ____ / ______

Name/signature of person obtaining assent: _____________________________

Date: ____ / ____ / ______
