# Supplementary material for: Adaptation and feasibility assessment of a school-based Suicide Prevention Intervention for Adolescents (SPREAD Study) in Nigeria: protocol for a cluster randomised controlled feasibility trial
Source: BMJ Open. 2026 Oct 1;16(10):e124182. doi: 10.1136/bmjopen-2026-124182 (PMC13629916; doi:10.1136/bmjopen-2026-124182)
Supplement: Supplementary file 5 [file bmjopen-2026-124182.reviewer_comments.pdf]

PEER REVIEW HISTORY

BMJ Open publishes all reviews undertaken for accepted manuscripts. Reviewers are asked to complete a checklist review form and are provided with free text boxes to elaborate on their assessment. These free text comments are reproduced below.

ARTICLE DETAILS

Title (Provisional)

Adaptation and Feasibility Assessment of a School-Based Suicide Prevention Intervention for Adolescents in Nigeria (the SPREAD Study): Protocol for a Cluster Randomised Controlled Feasibility Trial

Authors

Adewuya, Abiodun O; Olibamoyo, Olushola; Oladipo, Olabisi E; Atilola, Olayinka; Fatusi, Adesegun; Abodunrin, Ife O; Akeju, Kemi

---

VERSION 1 - REVIEW

**Reviewer**	**1**
**Name**	**Jacobsen, Pamela**
**Affiliation**	**University of Bath Department of Psychology, Psychology**
**Date**	**26-Aug-2026**
**COI**	**None**

Thank you for the opportunity to review this paper, which was a pleasure due to the clarity of writing and the comprehensiveness of the information presented. Trial protocols are usually lacking at least a few key details but with careful cross reference with the SPIRIT checklist provided I was satisfied that all key areas were included, relating to the design and conduct of the trial. The proposed trial is a cluster randomised trial of a suicide prevention programme, which is designed as a ‘universal’ intervention delivered in school settings for adolescents 13-17. The intervention (YAM – Youth Awareness of Mental Health) has already been developed and tested in European studies. The SPREAD study proposes to test this programme in Lagos (Nigeria) with some pre-trial work on adapting the intervention to be culturally appropriate to the Nigerian setting, using an established approach to make sure the ‘active ingredients’ of the intervention are retained intact in any revised version. The rationale for the need to use a cluster randomised design is well made (because the intervention is designed as a ‘whole school’ intervention and so randomising at the individual pupil level is inappropriate and could lead to contamination). The identification and randomisation of eligible schools is also clearly explained, in that eligible schools will be identified first, eligible pupils will be consented and complete baseline measures, and then

the school will be randomised (this is a stronger design methodologically compared to randomising schools first then recruiting to the 'cluster). The outcomes are clearly set out and are in line with the goals of the trial, which is a feasibility trial, laying the groundwork for a later efficacy trial. Progression criteria for each key feasibility outcome are set out in a traffic light system (Red/Amber/Green) to indicate what pre-set benchmarks would indicate a go, amend, or stop signal for a future trial.

I have no specific revisions to recommend to the authors, but as a general point, I wonder if the research team would be able to make the anonymised dataset available via a research depository which would provide reliable, enduring, and secure archiving for this very important study. This is highly preferable to suggesting the dataset would be available on reasonable request to the PI, as several empirical studies have shown that <10% of PIs actually respond to such requests meaning that the data is in effect not available at all (e.g. Gabelica, M., et al. (2022). "Many researchers were not compliant with their published data sharing statement: a mixed-methods study." Journal of Clinical Epidemiology 150: 33-41.)

VERSION 1 - AUTHOR RESPONSE

Reviewer 1

Comment: The reviewer did not request a specific methodological revision but recommended replacing the "available on reasonable request to the PI" approach with enduring repository-based archiving of the anonymised dataset.

Response: We agree with the principle of durable repository-based access and have revised the protocol accordingly. After publication of the primary trial results, a suitably de-identified participant-level dataset, data dictionary, and statistical analysis code will be archived in a recognised research data repository providing persistent identifiers and controlled-access functionality. Because this study concerns minors and includes sensitive information on suicidality, we have not committed to unrestricted public download of participant-level data. Access will instead require review of a data request, a Data Use Agreement, and any required ethical approvals, consistent with participant consent and applicable data-protection requirements. The repository identifier and access conditions will be reported in the primary results publication. This approach responds to the reviewer's concern about enduring availability while preserving appropriate safeguards for a sensitive adolescent dataset.

Change made: Main manuscript: Ethics and Dissemination, "Dissemination Plan"; Declarations, "Availability of Data and Materials". SPIRIT checklist items 29 and 31c.
